# A Microdeletion of Chromosome 9q33.3 Encompasses the Entire LMX1B Gene in a Chinese Family with Nail Patella Syndrome

**DOI:** 10.3390/ijms151120158

**Published:** 2014-11-05

**Authors:** Shujuan Jiang, Jiubin Zhang, Dan Huang, Yuanyuan Zhang, Xiaoliang Liu, Yinzhao Wang, Rong He, Yanyan Zhao

**Affiliations:** 1 Clinical Genetics, the Affiliated Shengjing Hospital, China Medical University, Shenyang 110004, Liaoning, China; E-Mails: jsjdreamer@163.com (S.J.); huangying_0301@163.com (D.H.); zyy.0526@163.com (Y.Z.); xiaoliang1981@126.com (X.L.); 2 Orthopedics Department, the First Affiliated Hospital, China Medical University, Shenyang 110001, Liaoning, China; E-Mail: zhangjiubin1988@126.com; 3 NO. 31 Middle School in Shenyang of Liaoning Province, Shenyang 110021, Liaoning, China; E-Mail: wangyinzhao@163.com

**Keywords:** gene deletion, *LMX1B*, MLPA, nail patella syndrome

## Abstract

Nail patella syndrome (NPS) is an autosomal dominant disorder characterized by nail malformations, patellar apoplasia, or patellar hypoplasia. Mutations within the *LMX1B* gene are found in 85% of families with NPS; thus, this gene has been characterized as the causative gene of NPS. In this study, we identified a heterozygous microdeletion of the entire *LMX1B* gene using multiplex ligation-dependent probe amplification (MLPA) in a Chinese family with NPS. The determination of the deletion breakpoints by Illumina genome-wide DNA analysis beadchip showed that the deletion was located in chromosome 9q33.3 and spanned about 0.66 Mb in size. This heterozygous deletion provides strong evidence for haploinsufficiency as the pathogenic mechanism of NPS.

## 1. Introduction

Nail patella syndrome (NPS; OMIM 161200) is an autosomal dominant disorder characterized by nail malformations, patellar apoplasia, or patellar hypoplasia. Additional skeletal abnormalities can be present that encompass the iliac horns, produce elbow dysplasia, cause progressive nephropathy, or produce primary open angle glaucoma; thus, it is apparent that the phenotype of this disease is variable among or within families [1,2,3,4,5,6].

In 1998, Dreyer *et al.* [7] showed that NPS is caused by mutations of the *LMX1B* gene. The involvement of this gene in NPS was subsequently confirmed by other studies [8,9]. *LMX1B* is one of the LIM-homeodomain proteins, which encode LIM-homeodomain transcription factors involved in pattern formation during development [10,11]. Previous studies have suggested that the *LMX1B* gene plays a pivotal role in the development of limb, kidney, eye, nervous system, as well as other organs or systems; these abnormalities are consistent with the phenotypes of NPS disease [7,8,12,13,14,15,16,17,18].

NPS is a rare hereditary disease with the incidence roughly estimated at 1 in 50,000 live births [19]. Mutations within the *LMX1B* gene have been detected in approximately 85% of families with NPS [1], including missense, nonsense, frameshift, splice-site mutations, small intragenic insertions/deletions, gross insertions/deletions, and complex rearrangements [7,8,9,20,21,22,23,24,25,26,27,28,29,9,20]. While most of the mutations were present in Caucasians, only a missense mutation c.742A>G (p.R248G) within the homeodomain of *LXM1B* has been reported to cause NPS in a Chinese family [30]. In this study, we first present the identification of a 0.66 Mb heterozygous microdeletion encompassing entire *LMX1B* and flanking the *MVB12B* and *ZBTB43* genes in a Chinese family with NPS.

## 2. Results

### 2.1. Clinical Manifestations

**Figure 1:**
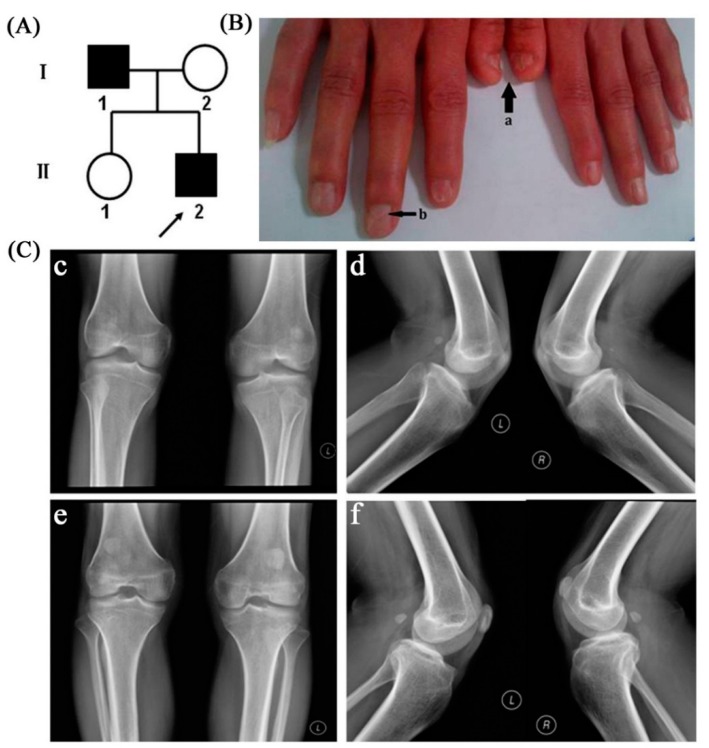
(**A**) Chinese pedigree with nail patella syndrome; patients are indicated by solid black, denoting the proband; (**B**) Clinical manifestation of the proband’s nails (**a**: short nail bed with longitudinal ridging; **b**: triangular lunula at the base of the nail); (**C**) Radiographic examination results of patients’ knee joint. The radiographs of proband’s knee joint showed severe bilateral patellar dysplasia (**c**,**d**); The radiographs of the father showed bilateral hypoplastic, superiorly misplaced patellae (**e**,**f**).

There were no other clinical abnormalities in the proband except for nail hypoplasia and patellar dysplasia. The nail abnormalities of the proband were prominent on both thumbs and the right index finger. They primarily manifested as nail bed shortening and longitudinal ridging; in addition, a typical triangular lunula was clearly visible in the proband’s nails (Figure 1B). Nail abnormalities of the father were subtle; they just manifested as a triangular lunula at the base of the nail. Radiographic examination results of the proband showed severe bilateral patellar dysplasia as his patella was obviously subnormal in size, while his father showed slight bilateral hypoplastic patellae that were displaced superiorly (Figure 1C). All subjects evaluated had normal renal function. There were no abnormalities of facial features, short stature, or elbow contractures in our patients, and there were no clinical abnormalities in other family members. The chromosomal analysis of the proband and his father revealed a normal male karyotype: 46, XY. Paternity was further confirmed by genotype analysis.

### 2.2. Genetic Analysis

**Figure 2:**
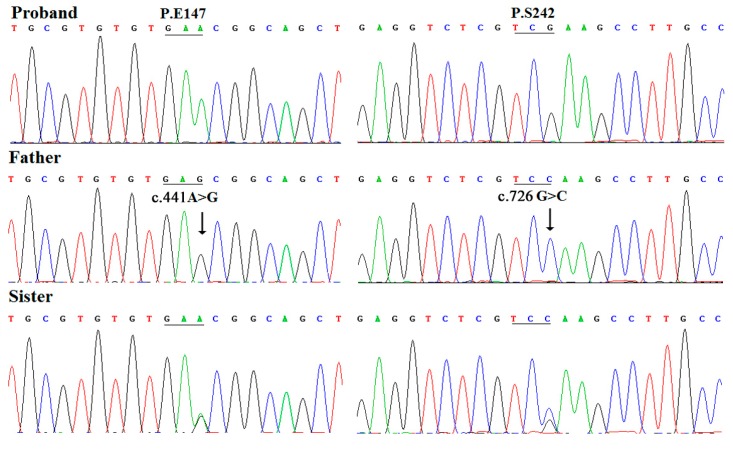
Chromatography of synonymous mutations of *LMX1B* gene in the family. The proband, his father, and elder sister, were wild type, homozygous at the 441 locus and heterozygous at the 726 locus.

Two hemizygous synonymous variants, c.441A>G (p.E147) and c.726G>C (p.S242), were detected in the proband’s father by direct DNA sequence analysis, these genetic alterations passed on to the proband’s normal elder sister as they were also identified (apparently heterozygous) in his sister (Figure 2). Notably, these two point mutations were not identified in the proband and his mother by DNA sequencing. These sequence results suggest a haploinsufficiency of *LMX1B* as the father’s synonymous variants were not passed on to the proband.

**Figure 3:**
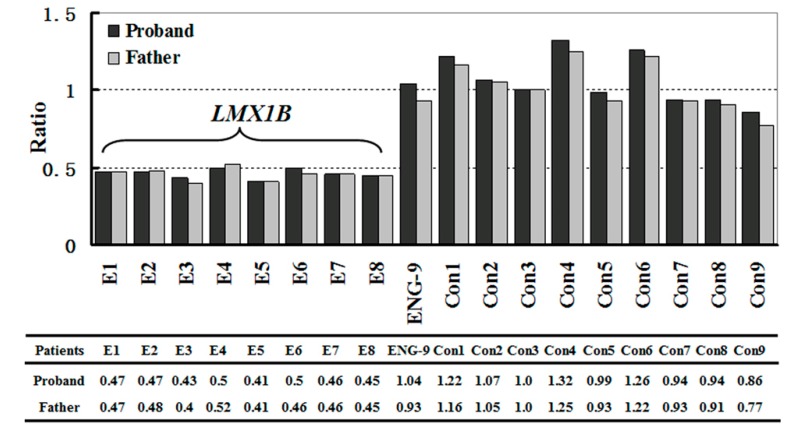
Results of MLPA analysis. A single-copy deletion of the entire *LMX1B* was detected in the proband and his father.

This hypothesis was confirmed by the results of MLPA analysis, which showed a single-copy deletion of the entire *LMX1B* (exons 1 to 8) in the proband and his father (Figure 3 and Figure S1). MLPA failed to detect deletions in the coding sequence of *LMX1B* in the proband’s mother and elder sister (Figure S1). These results confirmed that haploinsufficiency of *LMX1B* gene was the genetic pathogenic mechanism of this NPS family.

**Figure 4:**
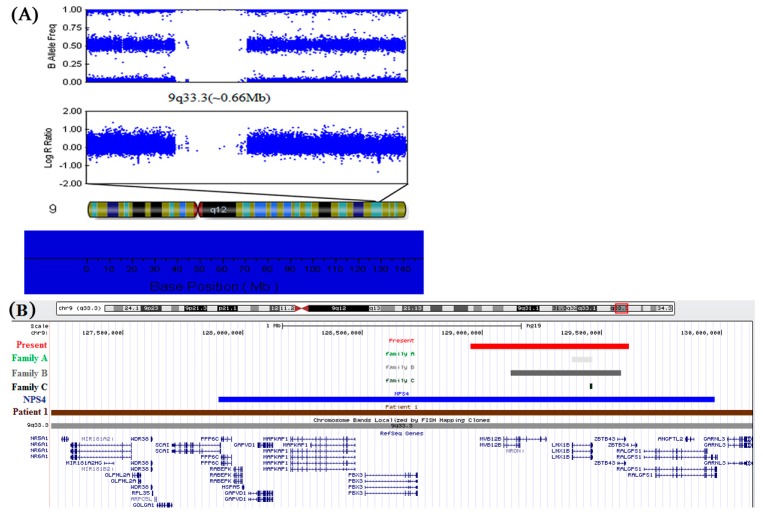
(**A**) The complete genome analysis of the proband. A 0.66 Mb deletion in chromosome band 9q33.3, between 128,952,700 and 129,613,085 bp, which was detected by using an Illumina genome-wide DNA analysis beadchip; (**B**) Enlargement of the 9q33.3–q34.11 region from the UCSC genome browser shows a comparison between the deleted segments, cytogenetics bands and RefSeq genes. Patient 1 from Schlaubitz *et al.* [32]: brown bar. Patients of families A, B, and C from Bongers *et al.* [25]: light gray to black bars. Patient NPS4 from Marini *et al.* [22]: blue bar. Patient in present study: red bar.

The complete genome analysis beadchip from Illumina was used to determine the breakpoints of the segmental deletion. The evaluation indicated a heterozygous deletion spanning from 128,952,700 to 129,613,085 in 9q33.3, which demonstrated the deletion to be 0.66 Mb in size [31]. This segmental deletion included the whole *LMX1B* gene, encoding a LIM-homeobox transcription factor as being the causative gene of NPS. In addition, it contained *MVB12B* and *ZBTB43* genes, which locate in the up and downstream of *LMX1B*, respectively (Figure 4).

## 3. Discussion

In this NPS family, patients displayed only nail and patellar dysplasia; no other clinical abnormalities were observed in the family. The nail abnormalities of the proband are prominent on both thumbs and the right index finger, primarily manifesting as nail bed shortening and longitudinal ridging; in addition, typical triangular lunula was clearly visible in the proband’s nails. Nail abnormalities of the father were subtle, just manifesting as a triangular lunula at the base of the nail. Radiographic examination of the proband showed severe bilateral patellar dysplasia, as his patellae were obviously subnormal in size, while his father showed slightly bilateral hypoplastic, higher than normal misplaced patella. The presence and severity of different NPS manifestations showed high variability at the individual, intrafamilial, and interfamilial levels. In addition to typical nail dysplasia and patellar apoplasia/hypoplasia, this disease could also manifest as elbow dysplasia, iliac horns, muscle dystrophy, progressive nephropathy, primary open angle glaucoma, attention deficit hyperactivity disorder, and symptoms of depressive disorder [1,2,3,4,33,34]. The phenotypic expression of NPS varies widely within and among families. This might be due to variable penetrance; however, other endogenous or environmental modifier factors could also be involved in the pathogenesis of this disease.

In the present study, two synonymous variants, c.441A>G (p.E147) and c.726G>C (p.S242), apparently hemizygous, were detected in the proband’s father and passed on to his elder sister without NPS. It is worth noting that these genetic alterations were not found in the proband and his mother by direct DNA sequence analysis. These results suggest that these synonymous substitutions could be single-nucleotide polymorphisms rather than pathogenic mutations and have no correlation with NPS [29]. Recently, the same synonymous mutation, c.726G>C (p.S242) of *LMX1B,* has been reported in a Korean Family with NPS [23]; the author could not demonstrate any segregation of this synonymous mutation with NPS. Our findings indicate that this genetic alteration of *LMX1B* was not pathogenic for this NPS family; thus, there must be other pathogenic mechanism for the observed phenomenon in this Korean family.

An increasing number of studies have attempted to elucidate the molecular pathogenic mechanism of NPS. In 1998, Dreyer *et al.* [7] demonstrated that NPS is the result of mutations within the *LMX1B* gene. Concurrently, Chen *et al.* [17] showed that *LMX1B*−/− mice exhibited limb and kidney defects similar to NPS. Moreover, Vollrath *et al.* [8] identified four mutations within *LMX1B* in four unrelated families with NPS and open-angle glaucoma (OAG). Since then, a large number of *LMX1B* mutations have been reported; however, no correlation in the range of severity of NPS symptoms has been reported among patients with missense, nonsense, frameshift, or splice mutations; furthermore, those with entire/partial gene deletions, strongly support haploinsufficiency for *LMX1B* as the mechanism of NPS [1,25]. This assumption is supported by the lack of any dominant-negative effect detected by *in vitro* experiments studying missense and truncation *LMX1B* mutations [30,35,36]. A study of *LMX1B*+/− mice showed diminished compensatory renal growth compared to the kidneys of *LMX1B*+/+ mice in which renal damage was induced by unilateral nephrectomy [18]. This result further supports the assumption that a critical dosage of *LMX1B* is critical for normal kidney development. The majority of mutations that have been identified are point mutations. Recently, Bongers *et al.* [25] identified two entire *LMX1B* gene deletions and one smaller partial *LMX1B* deletion (exons 3 to 8) in a series of eight unrelated Dutch families with classical features of NPS (Figure 4B and Table 1). Their finding strongly confirmed that loss of function is the main pathogenic mechanism of NPS in human. Marini *et al.* [22] and Schlaubitz *et al.* [32] identified two entire *LMX1B* gene deletions on chromosome 9q33.3–34.11 involving large regions (~2 and ~3.07 Mb) by using array-CGH (Figure 4B and Table 1). In addition to signs of NPS, both patients had facial anomalies, club feet, genital anomalies, and mental retardation. It is possible that other genes (except for *LMX1B*) deleted in these families could contribute to the etiopathogenesis of facial anomalies, club feet, genital anomalies, and mental retardation that were observed in these patients. In present study, a 0.66 Mb heterozygous microdeletion was identified in chromosome band 9q33.3 (128,952,700~129,613,085), encompassing the entire *LMX1B* and flanking *MVB12B* and *ZBTB43* genes in a Chinese family. This 0.66 Mb heterozygous deletion was first reported in NPS patients. In 2008, Bongers *et al.* [25] identified three different deletions in a series of eight unrelated families with classical features of NPS in whom no pathogenic *LMX1B* mutation was found by sequence analysis, as shown in Figure 4B, a deletion of exons 3–8 of *LMX1B* was found in family C, Further determination of the size of the genomic microdeletions revealed a deletion of the whole *LMX1B* gene in family A, whereas a deletion of the entire *LMX1B* and flanking *FAM125B* and *ZNF297B* genes was shown in family B which was similar to that of our patients [25]. However, it is uncertain whether these two deletions are identical because the location of the probes Bongers *et al.* [25] used were different than ours. The deletion was about approximately 0.44 Mb in length according to their probes’ position. Moreover, Bongers *et al.* [25] reported families revealed renal and extrarenal symptoms while our patients displayed only nail and patellar dysplasia. Despite this difference, our research can still further confirm the deletion of entire *LMX1B* as the pathogenic mechanism underlying NPS.

**Table 1:** *LMX1B* Deletions Reported.

Deletion	Size	Phenotype	Reference
Entire *LMX1B*	~0.66 Mb	NPS	Present study
Entire *LMX1B*	~82 Kb	NPS	Family A [25]
Entire *LMX1B*	~0.44 Mb	NPS	Family B [25]
Partial *LMX1B* (exon 3–8)	~5.4 Kb	NPS	Family C [25]
Entire *LMX1B*	~2 Mb	NPS, facial anomalies, club feet, mental retardation, genital anomalies	NPS4 [22]
Entire *LMX1B*	~3.07 Mb	NPS, facial anomalies, club feet, mental retardation, genital anomalies	Patient 1 [32]

## 4. Experimental Section

### 4.1. Subjects and Clinical Evaluation

This is a small family comprised of four members (Figure 1A). The proband is a 27-year-old-man who presented at our genetic clinic for nail hypoplasia. The proband’s father is also affected, while his mother and elder sister are normal. Detailed history and physical examination were carried out. Knee joints of the patients were assessed by radiographic examination. Cytogenetic analysis was performed to exclude a karyotype abnormality. Renal function was assessed by urinalysis and blood tests.

### 4.2. Sequencing of Genomic DNA

Genomic DNA was extracted from peripheral blood leukocytes using a DNA extraction kit (Watson Biotechnologies Inc., Shanghai, China), after obtaining informed consent. This experiment was approved by the ethical committee. Exons 1–8 of *LMX1B* were screened for mutations by DNA sequencing. Briefly, genomic DNA was amplified by PCR using the pair of primers (Table 2). PCR amplification was performed in 25 μL reaction volumes, containing 50 ng genomic DNA, 1× PCR buffer, 2× GC buffer, and 1 μM of each dNTP, as well as 1.5 μM·MgCl_2_ and 0.5 U Taq DNA polymerase (Takara, Dalian, China). After an initial denaturation at 94 °C for 5 min, the reactions were amplified for 35 cycles with denaturation at 94 °C for 45 s annealing at 61–68 °C for 45 s, and extension at 72 °C for 1 min; this was followed by a final extension at 72 °C for 10 min. DNA fragments were purified and subsequently sequenced and analyzed by the ABI PRISM 3730 DNA Analyzer (Applied Biosystems by Life Technologies., Carlsbad, CA, USA). The sequence data were analyzed by aligning with the reference sequences in NCBI (NC_000009 for *LMX1B*) using the DNAStar 5.0 (DNAStar., Madison, WI, USA) and BioEdit (Micro Focus., London, UK) software. Mutations or polymorphisms were identified according to the reference sequences.

**Table 2:** Primer Sequences for *LMX1B* Amplification from Human Genomic DNA.

Exon	Sense Primer	Antisense Primer	Product Size (bp)	Reference
1	TGACAAGCAGGTGACAGAGGA	CTGGCGATCACTCCAGGAGT	558	[5]
2	CCGAGGACTGGGACGGACTA	CTCTCGGAACCCTTGGAGCT	513	[5]
3	GGCAGGAGTGGCCTCTG	TCCAGGACACCCCAGCAAC	359	[6]
4 + 5 + 6	CCACGGCAGGTGTCAACAGA	GATGGCCTTGGTGGAAGGCT	1005	[5]
7 + 8	CTGAGCCTGGAGGAGGAGCT	GGGCACCGTATGGCTGT	1115	[5,7]

### 4.3. Multiplex Ligation-Dependent Probe Amplification (MLPA) Analysis

MLPA analysis was performed on the family members and two normal controls to identify large gene deletions or duplications in the *LMX1B* gene using the SALSA MLPA kit (P289-A2 *LMX1B*; MRC Holland, Amsterdam, The Netherlands). The P289-A2 *LMX1B* probemix contains 18 MLPA probes, including 8 probes for all exons of the *LMX1B* gene (exons 1–5, 6a, 7a and 8), 1 probe for ENG gene located on 9q34 and 9 reference probes, which were added to detect several different autosomal chromosomal locations. Hybridization, ligation, and amplification were performed according to the manufacturer’s protocol. Amplification products were detected using an ABI PRISM 3730 DNA Analyzer (Applied Biosystems by Life Technologies., Carlsbad, CA, USA) with LIZ500 (Applied Biosystems) as an internal size standard. The raw data were analyzed by using Coffalyser MLPA data analysis software (MRC Holland., Amsterdam, The Netherlands).

### 4.4. Whole Genome Copy Number Analysis

The IlluminaHumanOmniZhongHua-8 BeadChip (Illumina Inc., San Diego, CA, USA) was further used to determine the size of the sequence deletion in chromosome 9. The test was performed at Hunan Jiahui Genetics Hospital, Changsha, China. Experiments were conducted according to manufacturer’s protocol. Briefly, ~200 ng DNA was amplified, fragmented and hybridized onto the beadchip. After labeling, the beadchip was scanned using an Illumina BeadArray™ Reader (Illumina Inc., San Diego, CA, USA). Data were analyzed using the GenomeStudio software package (Illumina Inc., San Diego, CA, USA).

## 5. Conclusions

In this study, we identified a 0.66 Mb heterozygous microdeletion in chromosome band 9q33.3 encompassing the entire *LMX1B* gene and flanking *MVB12B* and *ZBTB43* genes in a Chinese family. This is the first report of a 0.66 Mb heterozygous microdeletion containing an entire *LMX1B* in NPS patients, which further confirmed the hypothesis that haploinsufficiency of *LMX1B* is the principal pathogenic mechanism of NPS in human.

## Supplementary Files

Supplementary File 1
