# Supplementary figures and images for: A Microdeletion of Chromosome 9q33.3 Encompasses the Entire LMX1B Gene in a Chinese Family with Nail Patella Syndrome
Source: Int J Mol Sci. 2014 Nov 5;15(11):20158–68. doi: 10.3390/ijms151120158 (PMC4264161; doi:10.3390/ijms151120158)

# Supplementary Information

Figure S1. Capillary electrophoresis pattern of MLPA analysis.

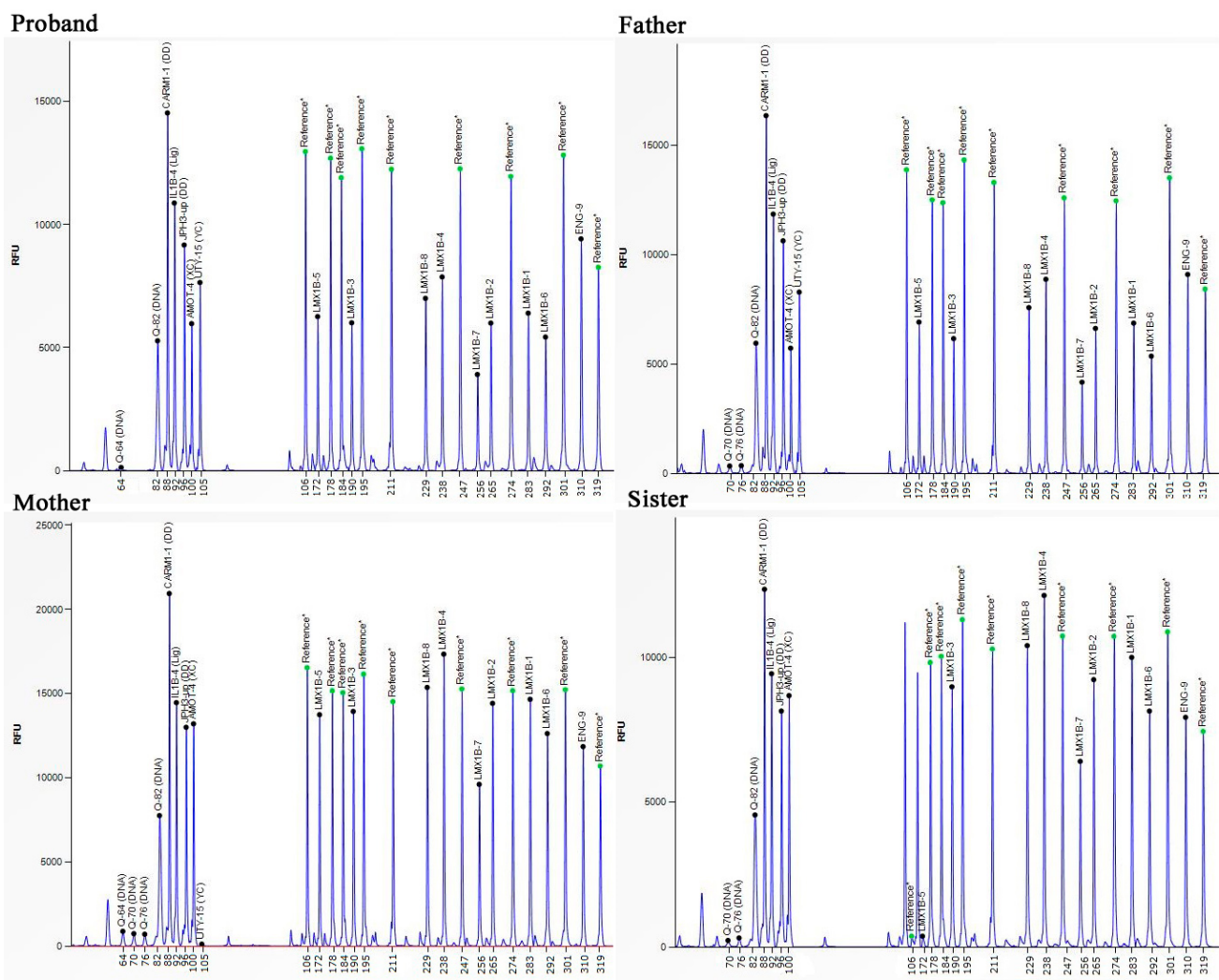

Supplement: Supplementary File 1 [file ijms-15-20158-s001.pdf]
